# NCOA4 Regulates Iron Recycling and Responds to Hepcidin Activity and Lipopolysaccharide in Macrophages

**DOI:** 10.3390/antiox11101926

**Published:** 2022-09-28

**Authors:** Cole A. Guggisberg, Juyoung Kim, Jaekwon Lee, Xiaoli Chen, Moon-Suhn Ryu

**Affiliations:** 1 Department of Food Science and Nutrition, University of Minnesota, St. Paul, MN 55108, USA; 2 Department of Biochemistry, University of Nebraska-Lincoln, Lincoln, NE 68588, USA; 3 Department of Food and Nutrition, Yonsei University, Seoul 03722, Korea

**Keywords:** ferritinophagy, ferritin, erythrophagocytosis, iron deficiency, heme, inflammation

## Abstract

Macrophages, via erythrophagocytosis, recycle iron from effete erythrocytes to newly developing red blood cells. Conversion of potentially cytotoxic levels of iron from its heme into nonheme form during iron recycling is safely accomplished via coordinated regulations of cellular iron transport and homeostasis. Herein, we demonstrate the roles and regulation of NCOA4 (nuclear receptor coactivator 4)-mediated ferritinophagy in macrophages after erythrophagocytosis using the mouse macrophage cell line J774 cells. Ferritin in J774 cells increased with the rise of nonheme iron by erythrocyte ingestion and declined when total cellular iron contents subsequently decreased. NCOA4, a selective autophagic cargo receptor for ferritin, was responsible for the control of cellular ferritin and total iron contents at the later stage of erythrophagocytosis. A hepcidin analog, which limits the flux of iron through iron-recycling by inhibiting iron export at the plasma membrane, repressed NCOA4 expression and led to accumulation of ferritin in the mouse macrophages. Transcriptome analyses revealed a functional association of immune response with NCOA4-dependent gene expressions, and we confirmed repression of *Ncoa4* by lipopolysaccharide (LPS) in J774 cells and the spleen of mice. Collectively, our studies indicate that NCOA4 facilitates cellular ferritin turnover and the release of iron by macrophages after erythrophagocytosis and functions as a regulatory target for molecular signals of systemic iron overload and inflammation. These identify macrophage NCOA4 as a potential therapeutic target for disorders of systemic iron dysregulation, including anemia of inflammation and hemochromatosis.

## 1. Introduction

Iron is essential to the mammalian cell due to the array of its biological functions. Iron in these cells functions as a cofactor for hundreds of metalloproteins, including those in DNA replication, electron transfer, and energy metabolism, and serves as a substrate for heme biosynthesis. Yet, excess iron can be toxic due to its redox-active nature [1]. Particularly, nonheme iron in the cytosol can lead to the production of hydroxy radicals, resulting in oxidative stress and cell damage when mishandled. Thus, cellular iron homeostasis and the labile iron pool must be tightly regulated for a balanced supply and demand. This is accomplished by coordinated expressions and regulated activities of proteins mediating the import, export, storage, and delivery of cellular iron.

At the organismal level, the primary consumer of iron is the erythron, where iron is used for production of heme. Human erythrocytes turn over every 120 days and are replaced through the supply of new red blood cells from erythropoiesis [2]. The large demand for iron by erythropoietic cells is met by the recycling of iron from senescent and effete red blood cells. Under normal steady-state conditions, iron recycled from erythrocyte turnover has been estimated to contribute up to 90% of the daily iron requirement for producing new red blood cell in humans [3]. Reticuloendothelial macrophages are the primary cell-types that carry out the recycling of heme into nonheme iron, and initiate the process by engulfing erythrocytes, i.e., erythrophagocytosis [3].

An individual human erythrocyte contains approximately 270 million molecules of hemoglobin (Hb), with each Hb composed of up to four heme iron molecules [4]. Thus, macrophages must be equipped to cope with the large expansion in its iron load after erythrophagocytosis. An important cytosolic protein for iron storage is ferritin. By forming a 24-mer spherical structure with ferritin L (FTL1) and ferritin H (FTH1) subunits, ferritin accommodates up to 4500 atoms of iron within its internal core [5]. Ferritin expression is translationally regulated by the labile iron pool via an iron response element (IRE) at the 5′-UTR of its transcripts. Expansion in the labile iron pool liberates IRE from iron regulatory protein (IRP) binding, which promotes ferritin translation [1]. Proteolysis of ferritin via lysosomal degradation has been proposed as the primary mechanism by which cells mobilize iron from ferritin [6,7]. The delivery of ferritin to lysosome is initiated by ferritinophagy, which involves the binding of a selective cargo receptor, NCOA4 (nuclear receptor coactivator 4) [8,9]. NCOA4-bound ferritin nanocages are transferred to autophagosomes for degradation and the release of their iron content.

Induction of ferritin expression by erythrophagocytosis has been observed in macrophages [10]. However, how ferritin iron is mobilized into the cytosol prior to its export from iron-recycling macrophages remains unclear. Iron accumulation in the spleen, a primary site for reticuloendothelial macrophages, has been observed in *Ncoa4* mutant models, suggesting a role of ferritinophagy in this process [9,11]. To gain a better understanding of the underlying mechanisms of ferritin regulation in macrophages, we employed an extensively validated in vitro model of erythrophagocytosis [10] and characterized the roles of ferritin and NCOA4 during iron recycling and their responses to molecules with physiological implications for organismal iron homeostasis and immune response.

## 2. Materials and Methods

### 2.1. Cell Culture and Iron Treatments

J774, clone E, and murine macrophages were acquired from Dr. Mitchell Knutson (University of Florida, Gainesville, FL, USA) and maintained at sub-confluency in AMEM containing 10% FBS (Sigma-Aldrich, Burlington, MA, USA; #F2442), 100 U/mL penicillin, and 100 μg/mL streptomycin. Cellular iron deficiency was produced by treatment of deferoxamine (DFO; Sigma-Aldrich #D9533) at a final concentration of 100 μM. Iron loading was achieved by supplementing the medium with 100 μg/mL of ferric ammonium citrate (FAC; Sigma-Aldrich #F5879).

### 2.2. In Vitro Erythrophagocytosis

The processes of erythrophagocytosis and red cell iron recycling were recapitulated in vitro using J774 cells as previously described [10]. Sheep erythrocytes in Alsever’s solution (HemoStat Laboratories, Dixon, CA, USA; #SBA050) were opsonized with rabbit anti-sheep IgG (MP Biomedicals #55806) by incubation at 37 °C for 20 min with 2.2 μg IgG per 1 × 10^6^ erythrocytes. Opsonized erythrocytes were washed by Alsever’s solution (Sigma-Aldrich #A3551), and added to the J774 at an approximate erythrocyte-to-macrophage ratio of 10:1. The time of erythrocyte treatment was considered 0 h (h) for time-course experiments. After 1.5 h of incubation, cells were washed with water to remove the erythrocytes remaining in medium by hypotonic lysis. Following two additional washes with PBS, cells were incubated in fresh growth medium until harvested at the designated times for analyses.

### 2.3. Hepcidin Mimic (PR73) and Endotoxin Treatments

PR73, a synthetic mini-hepcidin analog, was generously provided by Dr. Elizabeta Nemeth (University of California, Los Angeles, CA, USA). PR73 mimics the biological effects of hepcidin by blocking the efflux of cellular iron through ferroportin [12]. Erythrocyte-free macrophages were treated with 1 μM PR73 for 24 h. Considering the elevated ferroportin expression after erythrophagocytosis [10], a higher dose of PR73 at 4 μM was added when cells had been pretreated with opsonized erythrocytes. PR73 was added to cell culture 4 h after red cell treatments, and DMSO was added as a carrier control. J774 cells were treated with lipopolysaccharide (LPS; Sigma-Aldrich #L6529) as a model of endotoxemia at a concentration of 1.0 μg/mL for a duration of 24 h prior to harvest. To determine the effect of LPS in vivo, RNA was isolated from the spleen of LPS- or PBS-treated 16 week old male C57/B6 mice, which had been harvested, frozen, and stored from a previous study [13]. Each animal received LPS at 0.3 mg/kg bodyweight or PBS intraperitoneally 6 h prior to sacrifice. All animal experiments were approved by IACUC of University of Minnesota, and animals were handled following the National Institutes of Health guidelines.

### 2.4. Gene Silencing by siRNA Transfection

Gene silencing was achieved by liposome-mediated siRNA delivery using the HiPerFect transfection reagent (Qiagen, Venlo, The Netherlands; #301704). The negative control (Ambion, Waltham, MA, USA; #4390847) and NCOA4-specific siRNA (Ambion Silencer Select ID s77517) used in the present studies had been previously validated [14]. J774 cells were seeded at a density that maintains sub-confluency throughout subsequent culture periods. For a 6-well plate, cells were plated at 5 × 10^6^ cells in 500 μL of growth medium per well. Each siRNA was diluted to 300 nM with 500 μL of OptiMEM (Gibco, Waltham, MA, USA; #31985062), and then incubated with 15 μL of the HiPerFect reagent at room temperature for 5 min. Cells were treated with the siRNA-HiPerFect mixture and kept at 37 °C in 5% CO_2_. After 6 h of incubation, fresh growth medium was added to the transfected cells to yield a final siRNA concentration at 50 nM.

### 2.5. RNA Isolation, Reverse Transcription, and qPCR

Cells were harvested using a cell scraper and treated with TRI Reagent (Sigma-Aldrich, Burlington, MA, USA; #T9424) for RNA isolation. Total RNA was extracted via phase separation and RNA precipitation following the manufacturer’s instructions. Equal amounts of RNA were reversed transcribed to cDNA using the High-Capacity cDNA Reverse Transcription Kit (Applied Biosystems #4368814). qPCR was performed using the PowerUp SYBR Green Master Mix reagent (Applied Biosystems, Waltham, MA, USA; #A25742) with amplifications detected using a CFX Connect System (Bio-Rad). Melt curve analysis confirmed each reaction specificity. qPCR data was processed by the ΔΔCq method with *Actb* or *Tbp* as housekeeping genes for normalization. Primers validated and published elsewhere [15] were used for detection of *Hmox1*, *Hrg1*, and *Slc40a1* mRNA. For detection of other transcripts of interest, primers were designed using Primer-BLAST [16] and are provided in Table 1.

### 2.6. Protein Extraction and Western Blot Analysis

Cellular protein lysates were prepared using an NP-40 based lysis buffer [100 mM Tris-HCl (pH 7.5), 50 mM KCl, 0.1% NP40, 5.0% glycerol, water, and protease inhibitor cocktail (Roche, Basel, Switzerland; #11836170001)]. Total protein yields were determined using the Pierce BCA Protein Assay (Thermo Scientific, Waltham, MA, USA; #23225). For protein denaturation, equal amounts of protein were mixed with Laemmli buffer (Bio-Rad, Hercules, CA, USA; #1610747) and a reducing agent, 2-mercaptoethanol, and boiled for 10 min. Proteins were separated by electrophoresis using a Mini-PROTEAN TGX 4–20% polyacrylamide precast gel (Bio-Rad #4561096) in SDS-containing Tris-glycine running buffer (Bio-Rad #1610732). For native PAGE, protein samples were treated with Native Sample Buffer (Bio-Rad #1610738) and separated by the above precast gels in SDS-free Tris-glycine buffer (Bio-Rad #1610771) instead. For western blotting, proteins were then transferred to a nitrocellulose membrane using a Trans-Blot Turbo Transfer unit and RTA Transfer system (Bio-Rad #1704270). Protein transfer was confirmed by Ponceau staining. Membranes were blocked with a 5% nonfat milk-PBS solution. Primary antibodies were prepared in 5% nonfat milk-PBS-T and the following dilutions were used to detect each protein of interest: rabbit anti-ferritin at 1:1000 (Sigma-Aldrich, #F5012); anti-GAPDH at 1:2000 (Bio-Rad, #12004167); rabbit anti-NCOA4 at 1:1000 (Bethyl Laboratories, Waltham, MA, USA; #A302-272A); and rabbit anti-IRP2 at 1:1000 (Dr. Betty Leibold, University of Utah, Salt Lake City, UT, USA). Following primary incubation, proteins were probed with IRDye anti-rabbit secondary antibodies (1:10,000; Li-Cor #925-32211) and visualized using a Li-Cor Odyssey FC imager system (Li-Cor, Lincoln, NE, USA). Protein band intensity was quantified using the Image Studio Lite software (Li-Cor).

### 2.7. Cell Viability Assay

Cell viability and number of viable cells were determined using trypan blue exclusion and the Cell Counting Kit-8 (CCK-8; Sigma-Aldrich #96992). CCK-8 assay is a colorimetric method to assess the number of viable cells based on cellular dehydrogenase activity. J774 cells were plated in a 96-well plate at a density of 2000 cells/well and transfected as described above in a total well volume of 100 μL. Following 24 h of incubation, DFO (200 μM) was added to the medium and cells were incubated for another 18 h. Two hours prior to measuring the absorbance, 10 μL of CCK-8 solution was added to each well. Control wells were prepared without cells as a background correction for cell culture medium. The absorbance was measured at 450 nm and cell viability was determined as a percentage of the control.

### 2.8. Cellular Mineral and Heme Analyses

Cells were washed twice with PBS supplemented 10 mM EDTA to remove metals non-specifically bound to cell membranes. Acid-washed tubes were used to prevent contamination by external metal sources. Frozen pellets of known number of cells were processed and analyzed at the University of Nebraska-Lincoln for quantitative measures of mineral contents using inductively coupled plasma mass spectrometry (ICP-MS) following procedures previously described [17]. Briefly, cells were dissolved in 70% nitric acid at 70 °C for 3 h and then overnight at room temperature. Levels of major physiological ions were measured by ICP-MS (Agilent, Santa Clara, CA, USA; Model 7500 cs) coupled to a 96-well plate autosampler (Elemental Scientific Inc, Omaha, NE, USA). Cellular metal contents were normalized to total protein contents determined by BCA assays or cell numbers, and phosphorus contents were analyzed as a negative control. For heme assays, cell protein extract was prepared by solubilizing macrophages in Triton X-100 based lysis buffer [20 mM Tris-HCl (pH 7.5), 40 mM KCl, 0.5% Triton X-100, and water]. Heme contents were colorimetrically measured using the QuantiChrom Heme Assay Kit (BioAssay Systems, Hayward, CA, USA; #DIHM-250), following the manufacturer’s protocol. Absorbance at 400 nm was measured using a Synergy H1 plate reader (BioTek, Winooski, VT, USA) and normalized to total protein contents.

### 2.9. RNA-seq and Bioinformatic Analysis of Transcriptome Data

Total RNA for transcriptome analysis was isolated using the Direct-zol RNA Miniprep Kit (Zymo Research, Irvine, CA) following the manufacturer’s protocol. Subsequent procedures for RNA-seq were carried out at the University of Minnesota Genomics Center. The RNA Integrity Number (RIN) by an Agilent BioAnalyzer for every sample ranged from 7.8 to 8.5. Illumina sequencing libraries were constructed using TruSeq Stranded mRNA Library Prep Kit and sequenced on a NovaSeq 6000 instrument using a 150 paired-end (PE) flow cell at 20 million reads. The edgeR software package [18] was used for normalization and differential expression analysis of RNA-seq data. Core functional pathway analyses of the transcriptome data were conducted using the Ingenuity Pathway Analysis (IPA) software (Qiagen), and the gene set enrichment (GSEA) [19], g:Profiler [20], and Consensus PathDB Pathway tools [21]. Our RNA sequencing data have been deposited on Gene Expression Omnibus (GEO) under accession number GSE212772.

### 2.10. Statistical Analyses

Values are presented as mean ± SD. Significant difference between two groups were determined using Student’s t-test. For data of experiments with more than two treatment groups, one-way or two-way ANOVA with a Tukey’s post-hoc test was conducted for comparisons. Statistical significance was set at *p* < 0.05, and analyses were conducted using the JMP Pro software version 15.0.0 (SAS Institute, Cary, NC, USA).

## 3. Results

### 3.1. Post-Transcriptional Regulation of NCOA4 by Iron and NCOA4-Dependent Ferritin Turnover in J774 Macrophages

**Figure 1:**
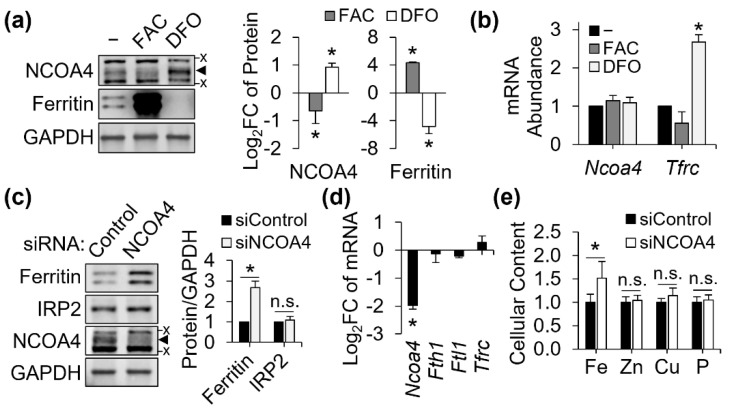
NCOA4 responds to cellular iron status and controls cellular ferritin and iron levels in J774 macrophages. J774 macrophages were treated with ferric ammonium citrate (FAC; 100 μg/mL) and deferoxamine (DFO; 100 μM) for 18 h to produce cellular iron overload and deficiency, respectively. Iron adequate cells (−) were without any iron treatments. For NCOA4 knockdown, cells were transfected with siRNA and cultured for 24 h. (**a**) NCOA4 protein abundance and its inverse relationship to cellular iron and ferritin levels (*n* = 3 independent experiments). (**b**) Responses of *Ncoa4* and *Tfrc* mRNA to iron treatments, normalized to *Tbp* (*n* = 3 independent experiments). (**c**) Effects of NCOA4 RNAi on ferritin and IRP2 protein abundance. Protein quantitation is normalized to GAPDH (*n* = 4 independent experiments). (**d**) Responses of iron homeostasis genes to *Ncoa4* silencing at the transcript level efficiency, normalized to *Tbp* (*n* = 4 independent experiments). (**e**) Cellular mineral contents normalized to cell counts (*n* = 5 biological replicates). All data presented as mean ± SD. *, *p* < 0.05. n.s., not significant. x, non-specific band. ◄, NCOA4 band.

We first assessed if NCOA4 is responsive to cellular iron status in our J774 cell culture model. Cellular iron overload by ferric ammonium citrate (FAC; 100 μg/mL) resulted in markedly increased ferritin protein abundance, while repressing NCOA4 (Figure 1a). Conversely, iron deficiency by deferoxamine (DFO; 100 μM), increased NCOA4 abundance with a concomitant decrease in ferritin protein abundance (Figure 1a). These findings demonstrate an inverse relationship between NCOA4 and ferritin expression in J774 macrophages. Notably, the transcript abundance of *Ncoa4* was not affected by either iron treatment, indicating a post-transcriptional mechanism for regulation (Figure 1b). *Tfrc* abundance confirmed the effectiveness of each iron treatment and demonstrated an active IRP/IRE system in J774 macrophages (Figure 1b). These findings indicate that NCOA4 is actively regulated by iron status in mouse macrophages and that regulated ferritinophagy may serve as a potential mechanism for controlling macrophagic iron storage and homeostasis.

To determine the role of NCOA4 in ferritin regulation and macrophage iron homeostasis, NCOA4 was knocked down by siRNA transfection. The siRNA-mediated gene silencing of NCOA4 was confirmed by repression in both protein (Figure 1c) and transcript abundance (Figure 1d) versus control levels. The loss of NCOA4 produced an increase in ferritin protein abundance that was on average 2.5-folds greater than control (Figure 1c). Typically, an increase in cellular iron is expected to expand the labile iron pool and thus repress IRP2 protein and *Tfrc* transcript abundance. However, NCOA4-deficient cells did not present any changes in these measures (Figure 1c,d), despite their higher contents of total iron relative to control cells (Figure 1e). This suggests that the retention of cellular iron by NCOA4 deficiency is not due to a change in the cytosolic labile iron pool but rather a different cellular compartment for iron storage, e.g., ferritin. Notably, only iron was affected by NCOA4 deficiency, indicating the specificity of NCOA4 for cellular iron regulation (Figure 1e).

### 3.2. NCOA4-Dependent Ferritin Turnover and Survival of Iron-Deficient J774 Macrophages

**Figure 2:**
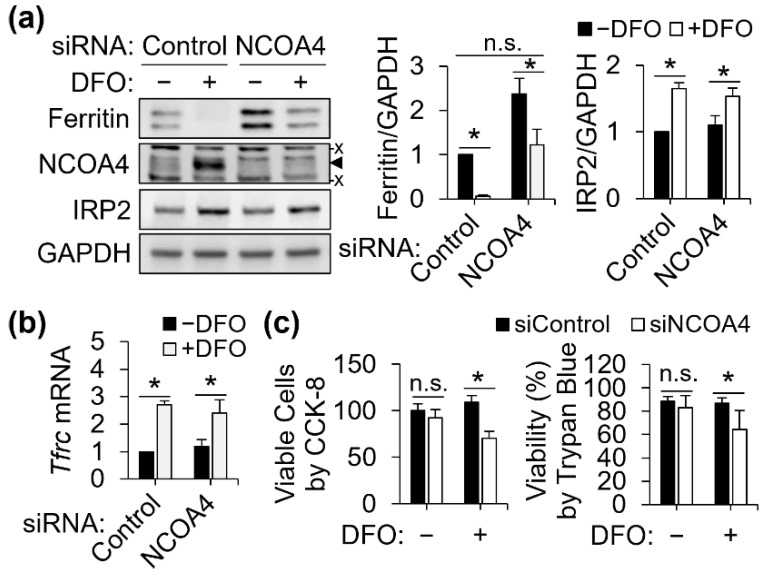
NCOA4 is required for the adaptation to cellular iron deprivation by J774 cells. J774 macrophages were treated with DFO (100 μM) for 18 h after 24 h of NCOA4 siRNA transfection. (**a**) Effects of NCOA4 and iron deficiency on ferritin and IRP2 protein abundance (*n* = 3 independent experiments). (**b**) Response of *Tfrc* mRNA abundance to iron restriction is not affected by NCOA4 depletion (*n* = 3 independent experiments). (**c**) Decrease in viable cell number by co-depletion of iron and NCOA4 by DFO and RNAi, respectively. Relative numbers of viable cells were determined using Cell Counting Kit 8 (CCK-8) and are shown in percentage of the average measure of cells with control siRNA without DFO treatment. Cell viability was determined by trypan blue exclusion (*n* = 4–5 biological replicates). All data presented as mean ± SD. *, *p* < 0.05. n.s., not significant. x, non-specific band. ◄, NCOA4 band.

The marked increase in NCOA4 expression by iron deprivation and its role in ferritin degradation suggest its involvement in the adaptation of macrophages to cellular iron restriction. To test this, J774 cells were treated with NCOA4 siRNA and DFO to restrict ferritin turnover and cellular iron import, respectively. By NCOA4 depletion, ferritin accumulation occurred in both iron-adequate and DFO-treated cells (Figure 2a). *Tfrc* transcript levels were similarly responsive to DFO in both control and NCOA4-deficient cells, confirming that the effectiveness of DFO for iron restriction were comparable in both control and NCOA4 siRNA-treated cells (Figure 2b). The repression in ferritin by DFO was also present in both control and NCOA4 siRNA-treated cells, presumably via increased IRP activity (Figure 2a). Both iron-adequate and -deficient cells presented accumulation of ferritin protein by NCOA4-depletion per se (Figure 2a). Notably, ferritin levels of NCOA4-deficient cells, when treated with DFO, became comparable to that of iron-adequate control cells.

Iron is essential for cell survival [22] and thus we assessed if the restriction of ferritin turnover by NCOA4 depletion impairs the cellular capacity to adapt to limited iron availability. Using the CCK-8 cell viability assay, the number of viable NCOA4-deficient cells was determined significantly lower than that of control cells when cellular iron import became restricted by iron chelation using DFO (Figure 2c). Notably, DFO treatment or NCOA4 deficiency alone did not significantly influence cell viability. These data identify NCOA4-dependent turnover of ferritin as an essential mechanism for J774 macrophages to survive cellular iron deficiency, and ferritin as an alternative iron source when its import becomes limited.

### 3.3. Cellular Heme and Non-Heme Iron Contents in J774 Macrophages after Erythrophagocytosis

**Figure 3:**
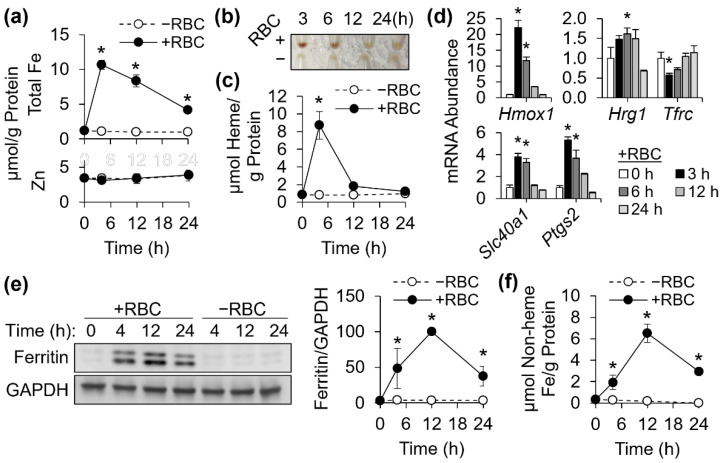
Cellular nonheme iron and ferritin protein contents transiently rise after erythrophagocytosis. J774 macrophages were treated with opsonized erythrocytes for 2 h to induce erythrophagocytosis, and then washed to remove residual non-ingested red cells. (**a**) Cellular contents of total iron and zinc after erythrophagocytosis, determined by ICP-MS and normalized to total protein contents (*n* = 4 biological replicates). (**b**) Cell pellet images of J774 cells treated with or without opsonized red cells. (**c**) Cellular heme contents of J774 cells after erythrophagocytosis, normalized to total protein levels (*n* = 4 biological replicates). (**d**) Regulation of gene transcripts related to iron transport (*Hrg1*, *Tfrc*, *Slc40a1*) and oxidative stress (*Hmox1*, *Ptgs2*) by erythrophagocytosis, normalized to *Tbp* (*n* = 3 biological replicates). (**e**) Immunoblotting of ferritin, and temporal expression pattern of ferritin abundance normalized to GAPDH after erythrophagocytosis (*n* = 4 independent experiments). (**f**) Cellular nonheme iron contents, calculated by subtracting normalized heme iron from total iron contents, after erythrophagocytosis (*n* = 4 biological replicates). All data presented as mean ± SD. *, *p* < 0.05.

The recycling of iron from ingested erythrocytes by macrophages requires the conversion of heme into nonheme iron, and its safe handling prior to export via ferroportin activity [3]. To assess the potential role of NCOA4 in iron handling during this process, J774 cells were treated with opsonized erythrocytes to induce erythrophagocytosis [10]. An acute increase in total cellular iron content, with a subsequent and gradual decline, by erythrocyte treatment was observed in these cells (Figure 3a). Notably, this temporal pattern was unique to iron and was not observed by measures of total cellular zinc (Figure 3a). J774 cell pellets featured an intense red color after 3 h of erythrocyte treatment, indicative of cellular accumulation of heme from ingested erythrocytes (Figure 3b). These were confirmed by quantitative assessments of heme iron (Figure 3c). After 12 h of erythrophagocytosis, both redness and heme measures of erythrocyte-treated cells became comparable to those of erythrocyte-untreated cells, indicating the conversion of heme iron into its nonheme form. In agreement with these are the expression of heme oxygenase-1 (*Hmox1*) and heme responsive gene-1 (*Hrg1*), which facilitates heme transport from the phagolysosome to the cytosol [15], and the repression of *Tfrc* mRNA early after erythrophagocytosis (Figure 3d). The gene transcript of ferroportin (*Slc40a1*) was upregulated by erythrophagocytosis as previously described [10]. *Ptgs2* transcript abundance has been measured as a marker of oxidative stress and ferroptosis [23] and was acutely upregulated at time-points of heme digestion (Figure 3d). Despite the rapid disappearance of heme, macrophage iron content remained elevated at 12 h (Figure 3a,c), indicating the retention of iron in a nonheme iron form. Thus, we measured ferritin by western blot analysis, which revealed a peak expression at 12 h after red cell ingestion (Figure 3e). Notably, the temporal expression pattern of ferritin in erythrocyte-ingested J774 macrophages were remarkably similar to that of nonheme iron content, calculated from the delta between total iron and heme iron contents of the cells (Figure 3f).

### 3.4. Facilitated Ferritin Turnover by NCOA4 after Erythrophagocytosis

**Figure 4:**
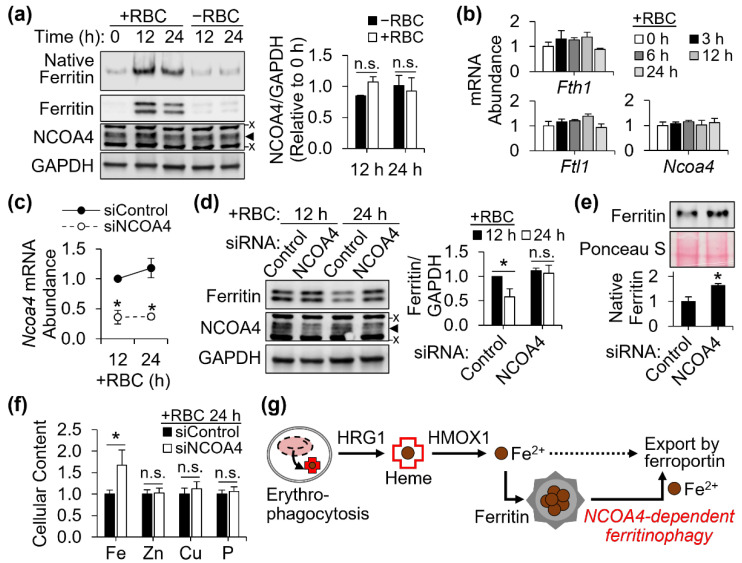
NCOA4 facilitates ferritin turnover and cellular iron release at later stage of erythrophagocytosis. J774 cells were treated with opsonized red blood cells at 0 h and washed at 2 h to remove non-ingested red cells. For NCOA4 depletion, cells were transfected with NCOA4 siRNA 24 h prior red cell treatment. (**a**) Expression of NCOA4 and native ferritin in erythrocyte-treated cells (*n* = 3 independent experiments). (**b**) Lack of changes in ferritin (*Fth1*, *Ftl1*) and *Ncoa4* transcript abundance by erythrophagocytosis, normalized to *Tbp* (*n* = 3 biological replicates). (**c**) NCOA4 knockdown confirmed by qPCR (*n* = 3 independent experiments) (**d**) Steady ferritin expression after erythrophagocytosis in J774 cells deficient of NCOA4. Protein abundance was normalized to GAPDH (*n* = 4 independent experiments). (**e**) Higher abundance of ferritin oligomers after 24 h of erythrophagocytosis in NCOA4-deficient cells, detected by western blot analysis of equal amounts of protein separated by native PAGE (*n* = 3 biological replicates). (**f**) Cellular mineral contents of erythrocyte-ingested J774 macrophages with or without NCOA4 deficiency, normalized to cell counts (*n* = 5 biological replicates). (**g**) Schematic illustration of the flux of iron and regulated ferritinophagy at different iron-recycling steps after erythrophagocytosis. All data presented as mean ± SD. *, *p* < 0.05. n.s., not significant. x, non-specific band. ◄, NCOA4 band.

Ferritin functions as an iron storage unit in its native oligomeric form, assembled by 24 ferritin H and L subunits. Western blot analysis of ferritin separated in its native form presented a temporal pattern similar to the expression of denatured ferritin protein, both with a peak at 12 h of erythrophagocytosis, followed by a decline in expression (Figure 4a). Ferritin transcripts, *Fth1* and *Ftl1* mRNA, did not reflect the changes in ferritin protein abundance after red cell ingestion by J774 cells, indicating a posttranscriptional control mechanism. As described earlier, NCOA4 mediates the delivery of ferritin to autophagosome to facilitate its lysosome-mediated turnover [8,9]. While neither the transcript nor protein abundance of NCOA4 were upregulated during the processing of ingested erythrocytes (Figure 4a,b), the loss of NCOA4 by RNAi (Figure 4c) led to the stabilization of ferritin expression between the timepoints of 12 and 24 h after erythrophagocytosis by J774 cells (Figure 4d). The accumulation of ferritin in NCOA4-deficient cells after 24 h of erythrocyte iron processing was also observed in the undenatured native form of ferritin (Figure 4e). The NCOA4-deficient mouse macrophages contained more iron than control cells when incubated for 24 h after treatment for erythrophagocytosis, while cellular contents of other minerals were not affected by the loss in NCOA4 (Figure 4f). Altogether, our findings propose that upregulation of ferritin allows macrophages to cope with the large amount of redox-active iron released after erythrophagocytosis and heme digestion, and NCOA4 facilitates the mobilization of ferritin iron prior to its export via ferroportin at later steps of macrophagic iron recycling (Figure 4g).

### 3.5. Repression of NCOA4 by Hepcidin Activity in J774 Mouse Macrophages

**Figure 5:**
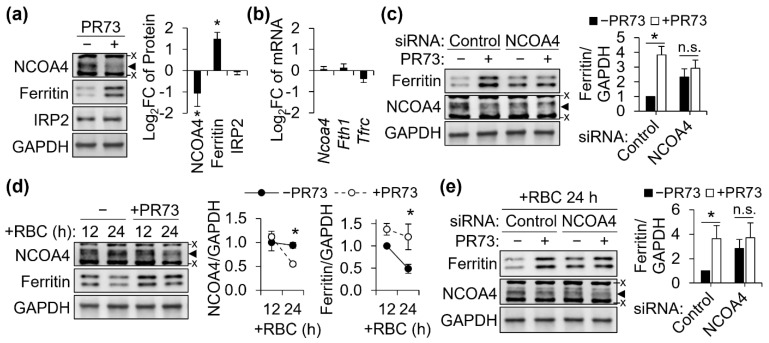
Hepcidin activity post-transcriptionally represses NCOA4 in J774 macrophages. J774 cells were treated with PR73, a minihepcidin analog, to mimic the biological effects of hepcidin in vitro. In experiments involving erythrophagocytosis, J774 cells were treated with opsonized red cells 4 h before PR73 treatment. For RNAi, cells were treated with NCOA4 siRNA 24 h prior to the red blood cell treatment. (**a**) Responses of NCOA4, ferritin, and IRP2 to PR73 (1 μM) in erythrocyte-free macrophages. Relative protein abundance was normalized to GAPDH (*n* = 3 independent experiments). (**b**) Lack of changes in *Ncoa4*, *Fth1*, and *Tfrc* mRNA abundance by PR73, normalized to *Tbp* (*n* = 3 independent experiments). (**c**) NCOA4 RNAi negates the increase in ferritin by hepcidin activity (1 μM). Ferritin quantification was normalized to GAPDH (*n* = 3 independent experiments). (**d**) PR73 (4 μM) repressed NCOA4 and prevented the facilitated turnover of ferritin between 12 and 24 h of erythrophagocytosis (*n* = 3 independent experiments). (**e**) Deficiency of NCOA4 represses the magnitude of change in ferritin by hepcidin activity on erythrocyte-ingested macrophages (*n* = 3 independent experiments). All data presented as mean ± SD. *, *p* < 0.05. n.s., not significant. x, non-specific band. ◄, NCOA4 band.

Cellular iron export by macrophages is a target of hepcidin due to their role in systemic iron homeostasis and innate immunity [24]. To assess the potential role of NCOA4 in macrophage iron regulation by hepcidin, J774 cells were treated with a synthetic hepcidin analog, PR73 [25]. Hepcidin activity mimicked by PR73 repressed NCOA4 abundance with a concomitant increase in ferritin protein abundance (Figure 5a). Transcript abundances of both *Ncoa4* and *Fth1* were unaffected by hepcidin activity, indicating post-transcriptional regulatory mechanisms (Figure 5b). Notably, IRP2 protein (Figure 5a) and *Tfrc* mRNA abundance (Figure 5b) were not affected by PR73, suggesting a mechanism offsetting the impact of hepcidin activity on the cytosolic labile iron pool. Thus, we tested whether downregulation of NCOA4 was sufficient for macrophages to handle the hepcidin effect by silencing NCOA4 prior to PR73 treatment. When J774 cells were deficient of NCOA4 and thus with higher basal ferritin levels, PR73 did not influence the ferritin abundance (Figure 5c). The downregulation of NCOA4 by hepcidin activity and diminished ferritin response to PR73 in NCOA4-deficient cells were also observed in cells treated with erythrocytes for 24 h (Figure 5d,e). These findings suggest that ferritin stabilization by NCOA4 repression mediates the adaptation of macrophages to cellular iron accumulation when cellular iron export is inhibited by hepcidin activity and thus protect cells from potential damage by oxidative stress from redox-active iron atoms.

### 3.6. Enrichment of Genes in Inflammatory Pathways by NCOA4-Responsive Genes in J774 Mouse Macrophages

**Figure 6:**
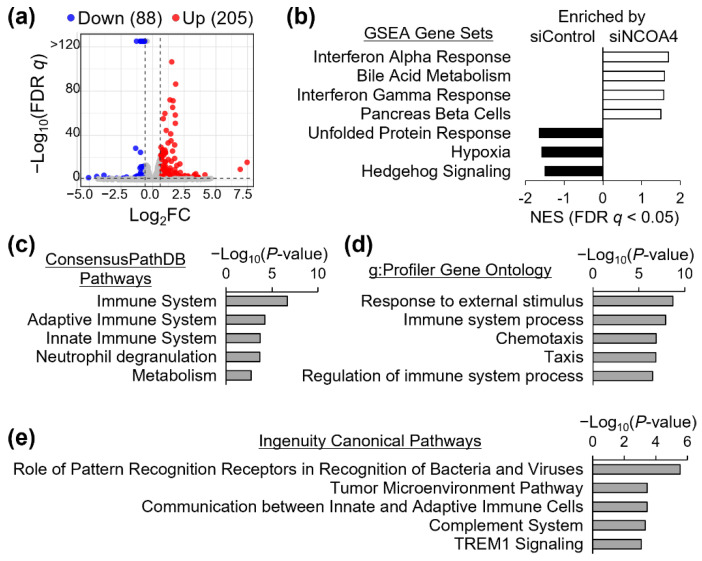
Transcriptome analyses of NCOA4 deficiency in J774 cells reveal gene responses functionally associated with immune system. Transcriptome of control and NCOA4 siRNA-treated cells were profiled via RNA-seq. (**a**) Total of 293 transcript responses were identified using |FC| > 1.5 and FDR-adjusted *q* < 0.05 as thresholds for differential expression and significance, respectively (*n* = 3 biological replicates). Among these, 88 were identified upregulated, while 205 genes were downregulated by NCOA4 deficiency. (**b**) Gene set enrichment analysis (GSEA) revealed enrichment of interferon response and hypoxia gene sets by genes with higher expressions in NCOA4 and control siRNA-treated cells, respectively. (**c**) ConsensusPathDB, (**d**) g:Profiler, and (**e**) Ingenuity Pathway Analysis tools commonly present over-representation of genes functionally annotated to immune system networks and pathways by NCOA4-responsive genes.

Our data reveal NCOA4 as a key regulator of ferritin iron in macrophages and thus a contributing element to cellular iron status. Previous work has demonstrated that cellular iron status can influence macrophage phenotypic polarization state, i.e., activation [26]. Therefore, to further ascertain the physiological implications of macrophage NCOA4, we characterized J774 cells deficient of NCOA4 using functional transcriptome analyses. Differential gene expression analyses via RNA-seq identified 293 responsive transcripts with |FC| > 1.5 and FDR q < 0.05 within the transcriptome of NCOA4-deficient cells (Figure 6a). Among these 205 were upregulated and 88 were downregulated by NCOA4 depletion (Figure 6a; Supplemental Data S1). GSEA identified four gene sets and three gene sets significantly enriched by genes upregulated by NCOA4 and control siRNA treatments, respectively (Figure 6b). The former included gene sets related to interferon α and γ-dependent responses, while gene sets of unfolded protein response and hypoxia were among the latter. Pathway enrichment analysis using the ConsensusPathDB and g:Profiler tools revealed enrichment of genes associated with immune system and response by differential expressions produced by NCOA4 depletion (Figure 6c,d). In agreement with these findings, the IPA software package for functional analyses of the transcriptome revealed over-representation of immunity-associated pathways by the list of genes affected by NCOA4 depletion (Figure 6e). Among the top 5 canonical pathways identified by IPA, two pathways were predicted to be activated by NCOA4 deficiency with activation z-scores above 2. These were pathways of ‘*role of pattern recognition receptors in recognition of bacteria and viruses*’ (z-score = 2.65) and ‘*TREM1 signaling*’ (z-score = 2.24). Pattern recognition receptors are a well-studied class of proteins, including toll-like receptors, which are expressed on the surface of immunes cells and involved in the innate immune response [27]. Triggering receptor expressed on myeloid cells-1 (TREM-1) is a receptor on the surface of monocytes and neutrophils that has been shown to modulate the TLR-4 cascade response to LPS [28].

### 3.7. NCOA4 Repression in Mouse Macrophages and Spleen by LPS

**Figure 7:**
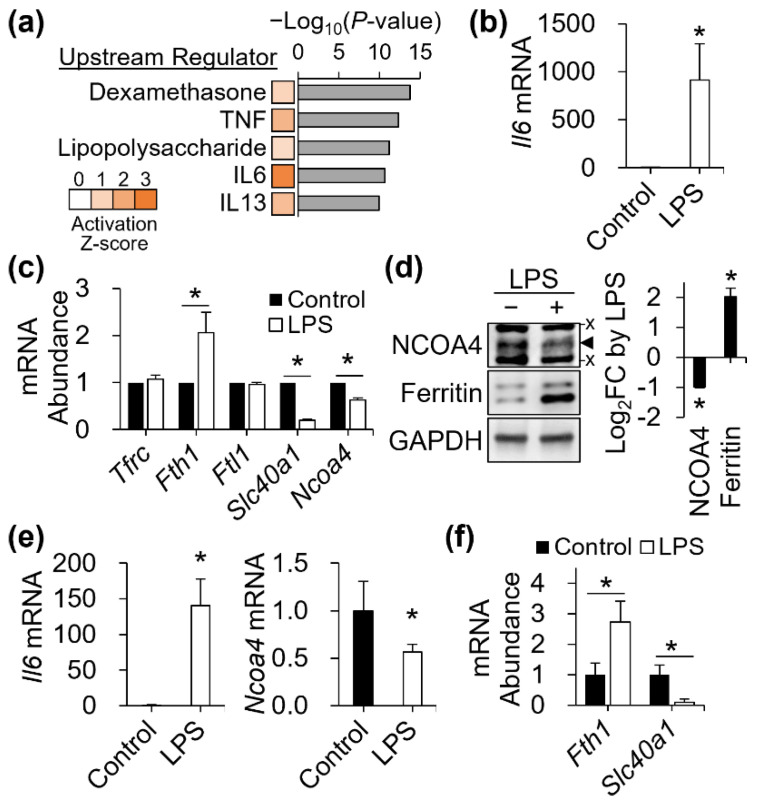
LPS downregulates NCOA4 at the transcript level in vitro and in vivo. (**a**) Top five most significant upstream regulators explaining the gene responses produced by NCOA4 deficiency in J774 cells. The positive activation score > 2 of IL6 predicts its activation in NCOA4-deficient cells. (**b**) Enhanced *Il6* transcript abundance by LPS in J774 macrophages (*n* = 4 independent experiments). (**c**) Responses of *Ncoa4*, *Fth1*, and *Slc40a1* transcripts to LPS in vitro (*n* = 4 independent experiments). (**d**) Repression of NCOA4 and elevation in ferritin proteins by LPS in J774 cells (*n* = 3 independent experiments). (**e**) Upregulation of *Il6* and downregulation of Ncoa4 transcripts in the spleen of male mice after 6 h of intraperitoneal LPS injection (*n* = 5 animals per group). (**f**) LPS-induced responses of splenic ferritin-H (*Fth1*) and ferroportin (*Slc40a1*) transcript abundance (*n* = 5 animals per group). All qPCR data were normalized to normalized to *Actb*. Data presented as mean ± SD. *, *p* < 0.05. x, non-specific band. ◄, NCOA4 band.

The upstream regulator analysis of IPA provides predicted upstream regulators that can lead to the observed differential expression profile. Among the highest ranked predicted upstream regulators for gene responses produced by NCOA4 deficiency were dexamethasone, LPS, and inflammatory cytokines, such as tumor necrosis factor (TNF), interleukin-6 (IL6), and IL13 (Figure 7a). Although each upstream regulator had a positive z-score, only IL6 reached the level of significant activation by a z-score above 2 (Figure 7a).

Our functional transcriptomic studies revealed an association between NCOA4 and inflammatory pathways and molecules. Previous studies demonstrate that activation toward inflammatory M1 macrophages involves coordinated regulation of iron genes leading to cellular iron sequestration [29]. Thus, we next tested whether macrophage NCOA4 expression is regulated by an inflammatory stimulus, LPS. Induction of *Il6* transcription confirmed the activation of J774 macrophages by LPS treatment (Figure 7b). LPS produced iron homeostatic gene responses towards a profile of cellular iron retention, including an increase in ferritin (*Fth1*) transcript for storage and a decrease in ferroportin (*Slc40a1*) for export. In agreement with these responses, NCOA4 was repressed by LPS at both the transcript (Figure 7c) and protein levels (Figure 7d). The response of ferritin to LPS was also measured at the protein level, with a particularly pronounced response in the lower molecular-weight band of the doublet (Figure 7d). The discrepancy in LPS-induced responses between the two ferritin bands may be explained by the lack of change in *Ftl1* transcript abundance, unlike *Fth1*, by LPS (Figure 7c).

The spleen is the organ hosting reticuloendothelial macrophages mediating erythrocyte iron recycling for steady state erythropoiesis. Thus, we next assessed if LPS can affect NCOA4 expression in the spleen of mice. Spleens of LPS-injected mice produced approximately two orders of magnitude higher levels of *Il6* transcripts after 6 h of injection versus PBS controls (Figure 7e). Similar to the responses of iron genes measured in LPS-treated macrophages in vitro, in vivo LPS treatment significantly reduced *Ncoa4* transcript abundance in the spleen (Figure 7e). Additionally, the in vitro responses of *Fth1* and *Slc40a1* transcripts to LPS were recapitulated by those in vivo (Figure 7f). These data identify a potential role of NCOA4 in directing cellular iron retention by macrophages under proinflammatory signals [29].

## 4. Discussion

NCOA4 is a cargo receptor protein initiating ferritinophagy by directly binding to ferritin [8,9]. This initial complex formation between NCOA4 and ferritin is promoted under cellular iron restriction where NCOA4 is stabilized [8,30]. When iron becomes abundant, iron functions as a bridging ligand between NCOA4 and an E3 ubiquitin ligase, HERC2, which leads to the post-translational downregulation of NCOA4 and thus ferritin iron accumulation [31]. Our studies identify the roles of NCOA4 in macrophage ferritin regulation, particularly for adaptation to iron restriction and when the flux of iron through iron recycling is under control by erythrophagocytosis, hepcidin activity, and inflammation.

The most prominent physiological role of macrophages in iron biology is to recycle heme iron from effete red cells and convert it into nonheme iron available to newly developing erythroid cells. Each red blood cell contains vast amounts of iron in the form of heme, and thus erythrophagocytosis may impose risk from an acute expansion in the cellular iron pool to macrophages. The current studies identify a transitional storage of iron within ferritin between heme digestion and the export of elemental iron by ferroportin. This is remarkably similar to the role of erythroid ferritin during terminal erythroid differentiation, where it functions as an intermediate form of iron between iron import and mitochondrial heme biosynthesis [14,31]. The heme molecules of phagocytosed erythrocytes are transferred to phagolysosomes, and then transported into the cytosol via a heme transporter HRG1 [15]. There, heme oxygenase 1 catalyzes heme into bilirubin, carbon monoxide, and ferrous iron [32], which is the form of iron transported out by ferroportin at the plasma membrane. Notably, we observed a delay in export of nonheme iron stemming from the digestion of heme introduced by erythrophagocytosis. Thus, ferritin iron as an intermediate form of cytosolic nonheme iron could function as a buffering mechanism for keeping the labile iron pool within a presumably manageable nontoxic range, as for the case of developing erythroid progenitors [14,31], after and before heme digestion and cellular export, respectively.

Recently, the physiological implications of facilitated ferritin turnover via ferritinophagy has been determined in vivo using mouse models of *Ncoa4* deficiency [9,11,33,34]. A series of them suggest a role of NCOA4 in controlling the rate of systemic iron recycling. First, splenic iron overload has been associated with microcytic anemia in mice with systemic germline deletions of *Ncoa4* [9,11,33]. Secondly, comparisons of hematological and iron indices between mice with erythron-specific and somatic *Ncoa4* knockout support contributions of both erythroid and nonerythroid NCOA4 to systemic iron supply [33], which would include macrophage NCOA4. Thirdly and most recently, wild-type mice with bone marrow transplanted from *Ncoa4*-null animals featured marked increase in splenic iron contents, particularly, when iron intake was restricted [34]. Our mechanistic cell culture studies further support the physiological importance of NCOA4 in ferritin regulation and iron recycling by macrophages (Figure 4g). Whether the majority or only a small fraction of iron from erythrophagocytosis enters the route of ferritin and ferritinophagy in macrophages remains to be determined, and studies on a macrophage-specific *Ncoa4* knockout model would be of particular relevance. However, considering the large contribution of iron recycling to the total daily iron requirement for new red cell production (over 90% for humans [3]), it is reasonable to predict that even a partial loss in this pool of iron supply would produce physiological consequences on systemic iron homeostasis and metabolism.

Similar to cellular iron balance, organismal iron distribution and homeostasis are regulated through an orchestrated control of iron utilization, acquisition, recycling, and storage. Central to the regulation of systemic iron homeostasis is the peptide hormone, hepcidin, which normalizes serum iron under systemic iron overload by inhibiting ferroportin activity on the plasma membranes of iron-supplying enterocytes, hepatocytes, and reticuloendothelial macrophages [2]. Ironically, the retention of iron within these cells by hepcidin may impose greater risk of oxidative stress and thus cytotoxicity when more iron is available due to organismal iron overload. Ferroportin is the sole iron exporter, transporting cytosolic iron across the plasma membrane. Thus, when hepcidin is present and cells lose their capability to remove excess iron via export, they must rely on an alternative mechanism for protection against iron-induced cytotoxicity. Of relevance is the sequestration of cellular iron into ferritin, which can be promoted by enhanced ferritin synthesis via the IRP-IRE regulatory system [5] and facilitated metalation of the storage protein via the iron chaperone activity of PCBP1 [14,35]. In addition to these, our studies indicate that conserving ferritin by restricting ferritinophagy could further support the capacity of cells to safely handle an expansion in intracellular iron when export becomes limited by hepcidin. Such cross-talk between ferroportin and ferritin mediated by NCOA4 has been previously demonstrated in ferroportin-overexpressing HEK293 cells [36] and is expected to provide protection against iron-induced organ or tissue failure by systemic iron overload.

Macrophages under signals of inflammation or infection become classically activated and polarize toward their M1 phenotype [37]. This induces a coordinated shift in iron metabolism genes to sequester iron within the macrophage as a function of nutritional immunity [38]. Increased hepcidin activity is also an integral element of nutritional immunity. These, however, may produce an iron-rich microenvironment within macrophages that could be favored by intracellular pathogens, such as *Salmonella* [39]. Thus, enhanced cellular capacity to sequester cytosolic iron would become physiologically and immunologically beneficial to the host under infection and could be accomplished by putting a brake on ferritinophagy as demonstrated herein. Notably, the repression of *Ncoa4* by LPS was measurable at the transcript level in both J774 macrophages and the spleen of mice, suggesting involvement of a molecular mechanism distinct from that by cellular iron status, which is primarily at the post-translational level [30,31].

Ferritin oligomers contain ferritin L and ferritin H subunits at various compositions under different cellular and physiological conditions. The enhanced production of ferritin by inflammatory stimuli have been demonstrated to be driven primarily by increasing ferritin H expression rather than that of ferritin L [26,40,41]. Ferritin H possesses ferroxidase activity which permits intracellular ferrous iron to be converted into ferric iron and stored in its biochemically inert form within ferritin [42]. While lacking ferroxidase activity, ferritin L contributes to the capacity of iron storage by ferritin [42]. Recently, ferritin H has been demonstrated to have another unique role in ferritin iron metabolism. Ferritin H but not L mediates the complex formation between NCOA4 and ferritin oligomers, which is required for the initiation of ferritinophagy [9,31]. Thus, the differences in the degree of ferritin H and L activation by inflammation could serve another physiological purpose. Ferritin complexes predominantly composed of ferritin H subunits would be more efficient in being targeted by NCOA4 and thus go through ferritinophagy. The selective upregulation of ferritin H under inflammatory conditions would be favorable for recovery from hypoferremia and to restore iron supply to meet erythropoietic or other physiological iron demands once infection or inflammation subsides.

Iron disorders and conditions leading to dysregulated iron affect millions of people, therefore the search for effective treatments to combat them is constant [2]. The present studies have clinical implications owing to the role of macrophages in systemic iron homeostasis. While hypoferremia induced by inflammation functions as an acute phase response [38], prolonged inflammation can lead to extended iron deficiency and thus anemia [2]. Notably, anemia of inflammation is the second most common form of anemia behind only iron deficiency anemia, affecting individuals with long-term infections, autoimmune disorders, cancer, chronic kidney disease, and many other inflammatory conditions [43]. It is characterized by normocytic or microcytic anemia and tissue iron accumulation. This is remarkably similar to the phenotypic presentation of *Ncoa4*-knockout mice [11,33]. Among the genetic disorders leading to iron dysregulation is hereditary hemochromatosis, characterized by tissue iron overload attributed to inadequate hepcidin activity and thus hyperabsorption of iron [44]. If untreated, hemochromatosis can lead to organ damage, cirrhosis, cardiovascular complications, diabetes mellitus, and increased risk of infections [44]. To date, the most common treatment strategy for this iron disorder remains phlebotomy [2], while recent efforts for the pharmaceutical application of synthetic hepcidin analogs against iron overload have been promising [12,25]. The current studies identify NCOA4 and ferritinophagy as upstream of the iron transfer through macrophage ferroportin, i.e., the supply of iron from iron recycling. Thus, pharmacologically modulating autophagy or more specifically NCOA4-mediated ferritinophagy [9,45] could be relevant to treatment of disorders of systemic iron dyshomeostasis, including anemia of inflammation and hereditary hemochromatosis.

## 5. Conclusions

This study details the roles and regulation of NCOA4 and ferritinophagy in J774 mouse macrophages, demonstrating the significance of the process in both cellular and systemic iron homeostasis. NCOA4 is imperative to the facilitated turnover of ferritin in the late stage of red cell iron recycling and a regulatory target of hepcidin and inflammatory stimulus. The repression of NCOA4 by iron overload and infection is expected to support the cellular capacity to safely retain iron in its chemically inert form and thereby protect cells against potential oxidative stress and microbial burden by intracellular pathogens. Thus, ferritinophagy may serve as a potential therapeutic target against disorders of systemic iron dysregulation, infection, and chronic inflammation. Further research employing animal models *Ncoa4*-deficient macrophages, dietary iron interventions, and pharmacological means targeting ferritinophagy would provide new insights on the clinical and nutritional implications of our findings at the organismal level and to human beings.

## Figures and Tables

**Table 1:** qPCR Primer Sequences.

Transcript	Primer Set	Sequence
*Ncoa4*	Forward	5’-AGCTAAGGCACCCAAGGCTA-3′
	Reverse	5’-CTTAGGGCCTCCTTTGCACG-3′
*Tfrc*	Forward	5′-TCACTTCCTGTCGCCCTATGT-3′
	Reverse	5′-AGAGTGTGAGAGCCAGAGCC-3′
*Fth1*	Forward	5′-CCACGTGACCAACTTACGCA-3′
	Reverse	5′-TCTCATCACCGTGTCCCAGG-3′
*Ftl1*	Forward	5′-GGAGCGTCTCCTCGAGTTTC-3′
	Reverse	5’-GAGATGGCTTCTGCACATCCT-3′
*Ptgs2*	Forward	5’-GCTCAGCCAGGCAGCAAATC-3′
	Reverse	5’-AGTCCGGGTACAGTCACACTT-3′
*Il6*	Forward	5’-CTCGGCAAACCTACTGCGTT-3′
	Reverse	5’-TGACCACAGTGAGGAATGTCCA-3′
*Actb*	Forward	5’-AGGAGTACGATGAGTCCGGC-3′
	Reverse	5’-AGCTCAGTAACAGTCCGCCT-3′
*Tbp*	Forward	5’-AGTTGTGCAGAAGTTGGGCT-3′
	Reverse	5’-TACTGAACTGCTGGTGGGTCA-3′

## Data Availability

The data presented in this study are available on reasonable request from the corresponding author.

## Supplementary Materials

The following supporting information can be downloaded at: https://www.mdpi.com/article/10.3390/antiox11101926/s1, Dataset S1: RNA-Seq Dataset of Control and NCOA4 siRNA-treated J774 cells.
